# QTL Mapping Reveals the Relationship between Pasting Properties and Malt Extract in Barley

**DOI:** 10.3390/ijms19113559

**Published:** 2018-11-12

**Authors:** Junmei Wang, Jianming Yang, Wei Hua, Xiaojian Wu, Jinghuan Zhu, Yi Shang, Meixue Zhou

**Affiliations:** 1 Institute of Crop and Nuclear Technology Utilization, Jinghuan, Hangzhou 310021, China; wangjunmei@zaas.ac.cn (J.W.); jmyang@163.com (J.Y.); huaweicau@hotmail.com (W.H.); wu.321@163.com (X.W.); jinghuanz@163.com (J.Z.); shangyi75@163.com (Y.S.); 2 Tasmanian Institute of Agriculture, University of Tasmania, Launceston, Tasmania 6250, Australia

**Keywords:** barley (*Hordeum vulgare* L.), quantitative trait loci (QTL), pasting property, malt extract, rapid visco-analyzer (RVA)

## Abstract

Pasting properties are important characteristics of barley starch from a processing standpoint. Many studies reported the close relationship between pasting properties and malting quality, especially malt extract. However, most conclusions were derived from the correlation between pasting properties and malting quality using a set of cultivars or breeding lines. In this study, a doubled haploid population of 150 lines from a cross between a Japanese malting barley and a Chinese feed barley was grown in four different environments (two sites × two years). Based on average values from all different environments, 17 significant quantitative trait loci (QTL) were identified for pasting properties. The genetic variance explained by these QTL varied from 7.0 to 23.2%. Most QTL controlling pasting properties were located on 1H, 2H, 5H, and 7H. Results confirmed the linkage between pasting properties and malt extract, with most of the QTL for pasting properties becoming nonsignificant when using malt extract as a covariate. Breakdown showed the closest correlation with malt extract. Molecular markers closely linked to the QTL can be used to select desired pasting properties to improve malting quality.

## 1. Introduction

Barley is an important primary source for the malting and brewing industries. Starch is the principal constituent of the total reserve of carbohydrates in barley grains, accounting for over 65% by weight of barley grain [1]. In barley endosperm, starch is deposited as granules, with two distinct populations of large (A-type) and small (B-type) granules [2]. The contents of both A- and B-type granule were higher in malting barley cultivars than in feed barleys [3]. During the brewing process, degradation products from starch are central in providing substrates for the fermentative phase, and the fermentative sugars contribute to the malt extract production. Therefore, the physicochemical properties of starch have a significant impact on the resultant malt quality and brewhouse performance [4].

Gelatinization is an important and functional characteristic of barley starch, essential for the complete enzymatic hydrolysis of starch during mashing [5]. During the gelatinization process, starch granules at progressively higher temperatures absorb water, begin to expand, and then rapidly swell. As a consequence of swelling, the viscosity of the solution increases, and nutrients are released [4]. The functional properties of starch are complicated traits, mainly controlled by genotypic factors [3,4,6,7,8]. Starch properties of barley depend on the composition and chemical structure of starch, such as the ratio of amylose and amylopectin, total starch levels, proportion of small starch granules, and mean large starch granule diameter in the mature barley grain [4]. Gelatinization properties are significantly correlated with the distribution of the amylopectin chain length [9]. Higher amylose content, higher first fraction of debranched amylopectin, and lower starch molecular weight lead to an increased gelatinization temperature and breakdown viscosity, but decreased peak and trough viscosity. The ratio of short chains to intermediate chains in amylopectin is negatively correlated with peak viscosity, breakdown, and gelatinization temperature of rice starch [10]. Several starch biosynthetic genes also show significant effects on starch properties [10], with the combination of *SSIIaj* (*indica* starch synthase IIa) and *Wxi* (*indica* Wx) with *SBEIIbj* (*japonica* starch biosynthase SBEIIb) and *SBEIi* (*indica* SBEI) alleles showing accumulative effects on lowering peak viscosity and gelatinization temperature. Stay-green-like traits have shown potential in ensuring food security, as the cultivar with such traits maintained starch biosynthesis and grain quality during drought conditions [11]. The properties of starch are under genetic control [10] but are also affected by environmental factors [9,12,13,14,15,16]. In general, high growth temperatures facilitate amylopectin crystallization, increase gelatinization temperatures, and delay the onset and lower the extent of swelling of granules when heated in water [10,13,14]. 

In order to optimize the selection of barley for malting, it is desirable to understand the genetic behavior of the properties of starch. A high heritability of starch granule traits was found based on genotype means, but the heritability was low in a parent-offspring heritability evaluation [8]. Flour pasting temperature was mainly controlled by additive effects with no significant dominance effects, and a high heritability (0.70–0.83) [17]. Quantitative traits loci (QTL) analysis provides a powerful tool for dissecting complex traits and identifying chromosome regions and molecular markers linked to these traits. The use of molecular markers associated with these traits can greatly improve selection efficiency. QTL affecting the starch granule traits have been reported on chromosomes 2H, 4H, and 5H [18], and QTL associated with flour pasting properties have been located on chromosomes 1H, 2H, 3H, and 7H [19].

The aims of this study were to identify QTL controlling flour pasting properties in a doubled-haploid (DH) population originated from a malting barley and a feed barley, and determine the relationship between pasting properties and one of the major malting quality traits, malt extract. 

## 2. Results

### 2.1. Pasting Properties for Parents and DH Lines

**Figure 1:**
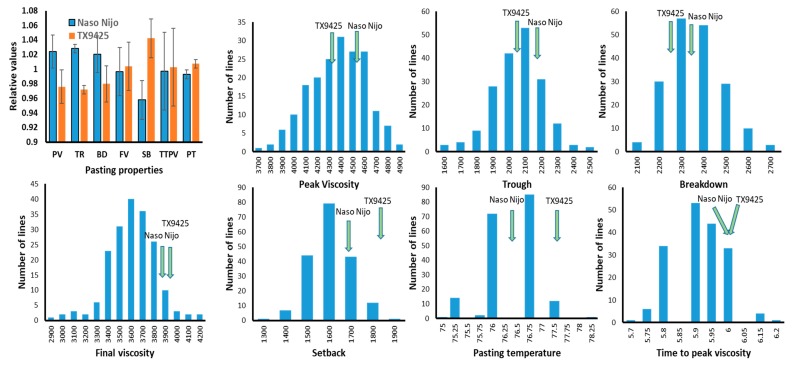
Distribution of different pasting properties in the DH population between the cross of TX9425 and Naso Nijo. Green arrows indicate the value s of two parents.

Mean values for the parents and the double-haploid population in each environment are shown in Table 1. Naso Nijo showed generally higher values for peak viscosity (PV), trough (TR), breakdown (BD), and setback (SB), while TX9425 had higher pasting temperature (PT) and longer time to peak viscosity (TTPV) (Figure 1). No significant differences in final viscosity (FV) and TTPV were found between the parents. Environments showed significant effects on pasting properties, with DH lines grown at Hangzhou, Zhejiang Province (HZ) having lower SB and FV, and shorter TTPV than those grown at Yancheng, Jiangsu Province (YC) (Table 1 and Table 2). DH lines showed a very significant difference in all pasting properties (Table 2). Normal distributions were found for most of the pasting properties (PV, TR, SB, FV, and PT), and transgressive segregations in the DH population were found for most of the pasting properties (BD and TTPV), indicating multiple genes controlling these parameters.

### 2.2. QTL Analysis for Pasting Properties

Based on average values from all different environments, three significant QTL were found to be associated with PV (Table 3, Figure S1). *QPv.NaTx-2H* was located on 2H with the nearest marker of 7213364S2, explaining 15.2% of the phenotypic variation. *QPv.NaTx-5H* was located on 5H with the nearest marker of 100000433D5, explaining 8.5% of the phenotypic variation. The third QTL, *QPv.NaTx-1H*, was located on 1H, explaining 7.4% of the phenotypic variation. The Naso Nijo allele increased PV on 2H, while that of TX9425 increased PV on both 1H and 5H. 

Three significant QTL associated with TR were identified. A major QTL (*QTr.NaTx-2H*) was located on chromosome 2H with the nearest marker being 3267579D2 (55.56 cM), explaining 21.3% of the phenotypic variation (Table 3). Naso Nijo contributed the allele for increasing TR for this QTL. Two other QTL, *QTr.NaTx-5H* and *QTr.NaTx-7H*, were identified on 5H and 7H, determining 10.3% and 7.4% of the phenotypic variation, respectively. TX9425 contributed the allele for increasing TR for both QTL.

Only one significant QTL (*QBd.NaTx-2H*) was identified for BD. This QTL was located on 2H with 9772745S2 being the closest marker, determining 19.5% of the phenotypic variation. Naso Nijo contributed the allele for increasing BD values. Three significant QTL for FV (*QFv.NaTx-2H*, *QFv.NaTx-7H*, and *QFv.NaTx-5H*) were located at the same position as those for TR. 

Three QTL (*QSb.NaTx-1H*, *QSb.NaTx-2H*, and *QSb.NaTx-7H*) were found to be associated with SB. *QSb.NaTx-1H* was located on 1H with the nearest marker being 3261249S1, explaining 19.2% of the genetic variation. *QSb.NaTx-2H* was located on 2H with the nearest marker being 3986974D2, explaining 10.8% of the genetic variation. *QSb.NaTx-7H* was located on 7H with the nearest marker being 3262448D7, explaining 7.3% of the genetic variation (Table 3). The Naso Nijo allele increased SB for the QTL on 1H and 2H, but decreased SB for the QTL on 7H. 

Two significant QTL (*QTtpv.NaTx-2H* and *QTtpv.NaTx-7H*) were found for TTPV (Table 3). *QTtpv.NaTx-2H* was located on 2H with the nearest marker being 3432484D2, explaining 11.8% of the genetic variation. *QTtpv.NaTx-7H* was located on 7H with the nearest marker being 3269237D7, explaining 9.2% of the genetic variation. The TX9425 allele increased TTPV in both QTL.

One major QTL (*QPt.NaTx-2H*) was identified for PT. This QTL was located on 2H with the nearest marker being 6429430S2, explaining 23.2% of the phenotypic variation. Another tentative QTL (*QPt.NaTx-1H*) was identified on 1H, explaining 7.0% of the phenotypic variation. The TX9425 allele increased PT in both QTL (Table 3). 

### 2.3. Correlations Between Pasting Properties and Malt Extract

As expected, most of the pasting properties were closely related (Table 4). Pasting properties also showed a significant correlation with malt extract. BD showed the highest correlation with malt extract determined according to the official method of the European Brewery Convention (EBC, 1998) (r = 0.50), followed by PV (r = 0.42), TTPV (r = −0.31), PT (r = −0.28), and TR (r = 0.21). SB was not significantly correlated with malt extract. 

## 3. Discussion

### 3.1. Multi-Environments Are Crucial for Determination of Flour Quality

Varying environments affect various quality traits, including barley malting quality [20]. Flour pasting properties can also be significantly affected by environments [21,22] through changed kernel size, and protein and lipid contents [23]. In this study, flour pasting properties were also affected by the environment (Table 2), with QTL detected for different parameters showing some differences between the different environments. For example, *QPv.NaTx-2H* was identified in YC07, HZ10, and YC10 trials. The QTL on 1H (*QPv.NaTx-1H*) was found in both HZ10(PV3.1) and YC10(PV4.1) trials. When using average data from all four environments, a new minor QTL (*QPv.NaTx-5H*) was identified. Some more QTL were also identified in individual trials (data not shown). Therefore, data from multi-sites/years are needed for accurate phenotyping of quality traits, including pasting properties.

### 3.2. Flour Pasting Properties Showed Close Relationship with Malt Extract

As one of the most important quality traits of malting barley, malt extract is affected by various factors, including starch content and composition, starch granule size, and physiological properties [4,20,24,25,26]. Starch pasting properties have been reported to be important contributors to malt extract. Good malting quality is associated with low TTPV and FV, but not necessarily with low peak viscosity or peak area [24]. Stuart et al. [7] also reported that malt extract was closely and negatively correlated with PT, as well as TR, PV, and TTPV. Starch with lower PT is more accessible in malted barley and, more importantly, the granules swell more easily under mashing conditions and are thus more susceptible to enzyme hydrolysis [7,24]. Most of the studies are based on starch. When using whole barley flour, the pasting properties are determined by various factors such as starch concentration and composition [26], and protein, lipid, and beta-glucan contents [27,28]. However, later studies have confirmed that the pasting properties of whole grain flour are closely related to malt extract [25], with high PV, TR, BD, and FV, and low TTPV and PT being related to high malt extract. Pasting properties of whole barley flour are also correlated with the uptake of water by seeds, which is an essential and initial step towards germination in the malting process [29] and fermentability [30].

**Figure 2:**
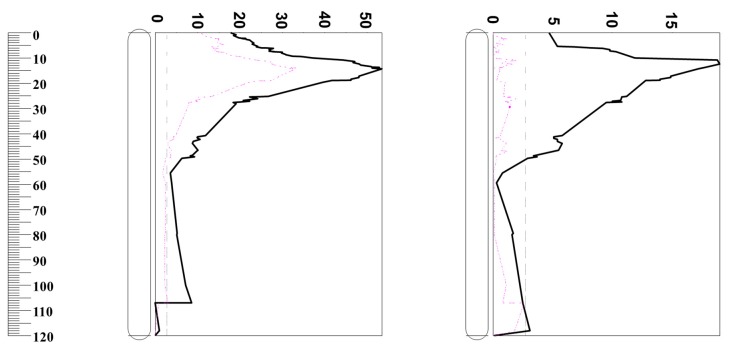
Percentages of phenotypic variation determined by QTL on 2H. **Left**: Malt extract with (red-dashed line) and without (black-solid line) breakdown as a covariate. **Right**: Breakdown with (red-dashed line) and without (black-solid line) malt extract as a covariate.

QTL analysis has been successfully used to reveal the linkage between different traits [31,32,33]. In this experiment, QTL analysis for malt extract following the European Brewery Convention (1998) was conducted using different pasting properties as covariates. As shown in Table 5, TR, FV, and SB had little effect on malt extract. When using PV, TTPV, and PT as covariates to analyze QTL for malt extract, the percentage of phenotypic variation explained by the QTL decreased from 53.5% to 41.9%, 44.1%, and 45.8%, respectively. BD was the most important trait contributing to malt extract. When BD was used as a covariate, the percentage of malt extract variation explained by the QTL decreased from 53.5% to 33.2% (Table 5 and Figure 2). High values of BD are likely to be associated with a high degree of collapse of swollen starch granules (low trough viscosity) corresponding to a greater release of solubilized starch capable of re-association during the cooling portion of the RVA profile [34]; thus, this will increase the malt extract as β-amylase does not attack whole intact starch granules, rather, it rapidly hydrolyzes a high proportion of solubilized starch and starch dextrins to maltose.

The association between malt extract and pasting properties was further confirmed by using malt extract as a covariate to analyze QTL for pasting properties. Table 3 shows that the contribution of most of the QTL to pasting properties became nonsignificant when using malt extract as a covariate. For those which were still significant, the percentages of variation determined by the QTL was significantly reduced. The European Brewery Convention (1998) defined one of the methods for measuring malt extract. When a different method was used, for example, the Institute of Brewing Method (Martin and the Analysis Committee, 1979), which involves a high temperature infusion mash, the results of malt extract can be different and may thus lead to a different relationship between pasting properties and malt extract.

### 3.3. QTL for Malt Extract Are Associated with Flour Pasting Properties

Pasting properties can be used as selecting criteria for malt extract. However, even though the measurement of pasting properties is relatively easy and needs only about 5 g of seeds, given that most of the flour components are affected by the environment [12,19,35,36], flour pasting properties could be affected by environmental factors, which was also confirmed in the current experiment. The identification of molecular markers linked to pasting properties can make the selection more effective in a breeding program. There are very limited reports on QTL for barley pasting properties. In our previous research [19], we mapped many QTL for pasting properties from a cross between Yerong (an Australian feed barley) and Franklin (as Australian malting barley). Comparing QTL from the present study with those in the previous one, only *QTr.NaTx-2H*, *QSb.NaTx-2H*, and *QTtpv.NaTx-2H* are located at positions similar to those of *QTr.YeFr-2H*, *QSb.YeFr-2H*, and *QTpv.YeFr-2H*, respectively. *MLOC_60943.2* could be the candidate gene that codes for endo-1,4-beta-xylanase, an important enzyme hydrolyzing the backbone of heteroxylan and releasing soluble polysaccharides [37]. All other QTL for pasting properties are different from those previously reported. These results are not without expectation. In the previous population [19], the malting barley variety, Franklin, contributed positive alleles for malt extract on 1H (close to centromere) and the long arm of 7H [38]. Several QTL for pasting properties were at similar positions [19] to those for malt extract [38]. However, in the current population, the malting barley variety, Naso Nijo, contributed positive alleles on the short arm of chromosome 2H [20], where several QTL for pasting properties are located.

## 4. Materials and Methods 

### 4.1. Plant Materials and Field Experiments

A total of 150 doubled-haploid (DH) lines were produced from the F1 of the barley cross between TX9425 and Naso Nijo by the anther culture method [39]. TX9425 is a Chinese two-rowed feed variety, which shows shorter plant height and good tolerance to stresses [40,41]. In contrast, Naso Nijo is a Japanese two-rowed malting barley with good agronomic traits but less tolerant to stresses. The two parents also showed significant differences in pasting properties [17]. 

All the DH lines and parents were grown in Hangzhou, Zhejiang Province (HZ, 30.25° N, 120.17° E) and Yancheng, Jiangsu Province (YC, 33.38° N, 120.12° E) in two different growing seasons, 2007–2008 (07) and 2010–2011 (10). A total of 150 vigorous seeds of each line or variety were sown in 2-m rows with 0.25-m row spacing. All experiments were arranged in a randomized complete block design with three replications. The soil type was silt-loam with medium fertility. All plots were supplied with 150 kg/ha N, 150 kg/ha P, and 80 kg/ha K as base fertilizer before sowing. No further fertilizer was applied to the plots. Fungicides were not required as no severe diseases were observed. Hand weeding was undertaken when needed. On maturity, the grains of each line or variety were harvested and stored in a room at −4 °C for further analysis.

### 4.2. Measurement of Pasting Properties

The whole barley grains were ground to pass a 1 mm screen. Ground flour (4.0 g) was made into a slurry with 0.1 M silver nitrate solution (25 mL). The use of 0.1 M silver nitrate solution aimed to inactivate different enzymes that may be produced during storage [6]. The pasting properties of the slurry were determined with a rapid visco-analyzer (RVA-TecMaster, Perten, Sweden) using a stirring speed of 960 rpm for the first 10 s and 160 rpm for the remainder of the test. The temperature was programmed to rise from 50 °C to 95 °C in 3.7 min, to remain for 2.5 min, to cool to 50 °C in 3.8 min, and to remain for 2 min. RVA measurements were reported in cP, min, and °C. The RVA measurements were as follows [25]: peak viscosity (PV), highest viscosity during heating; time to peak viscosity (TTPV); trough (TR), lowest viscosity after cooling started; breakdown (BD), peak viscosity minus trough; final viscosity (FV), maximum viscosity after the temperature had returned to 50 °C; setback (SB), final viscosity minus trough; pasting temperature (PT), temperature when the viscosity reaches 10% of the peak viscosity.

### 4.3. Micro-Malting and Malt Extract Analysis

The barley grains were screened through a 2.2-mm sieve, and 200-g grain samples of each line were micro-malted in an automatic micro-malting system (Joe White Micro-malting Systems, Adelaide, Australia). Malt extract was determined according to the official method of the European Brewery Convention (EBC, 1998). This method is similar to that of the Institute of Brewing (IoB), with the extract following the EBC method being about 1.1% higher than that following the IoB method due to 0.2 mm (fine grind) for EBC compared to 0.7 mm (course grind) for IoB [42,43]. The average data from four different environments (two different sites × two growing seasons) [20] were used to analyze the relationship between pasting properties and malt extract.

### 4.4. Map Construction

Genomic DNA was extracted from the leaf tissue of 3-week old seedlings, based on a modified CTAB (cetyl trimethylammonium bromide) method described by Stein et al. [44]. DH lines and the two parental varieties were genotyped with DArTSeq (a restriction enzyme-mediated genome complexity reduction approach with subsequent NGS) (http://www.diversityarrays.com/dart-application-dartseq). Due to the large number of DNA markers [~30,000 SNP (single-nucleotide polymorphism) and DArTSeq markers], markers with the same positions or with greater distortion and missing data were removed from the map construction. These markers were combined with previous genotypic data [DArT and SSR (simple sequence repeats markers)] [39,45]. A total of around 2500 markers were selected to construct the genetic map.

### 4.5. QTL Analysis

A genetic linkage map produced from the TX9425/Naso Nijo DH population using over 2500 markers was used for QTL analysis. The QTL analyses were based on the mean of the three replications from each site and year. The software package MapQTL6.0 [46] was used to detect QTL, which were first analyzed by interval mapping (IM). The closest marker at each putative QTL identified using interval mapping was selected as a cofactor, and the selected markers were used as genetic background controls in the approximate multiple QTL model (MQM). Logarithm-of-the-odds (LOD) threshold values, applied to declare the presence of a QTL, were estimated by performing genome wide permutation tests using at least 1000 permutations of the original data set for each trait, resulting in a 95% LOD threshold of around 3.0. To determine the effects of pasting properties on the QTL for malt extract, QTL for mat extract were re-analyzed by using different pasting properties as covariates. The percentage of the variance explained by each QTL (R^2^) was obtained by using restricted MQM mapping. Graphical representation of linkage groups and QTL was carried out using MapChart 2.2 [47].

## 5. Conclusions

Many QTL were identified for different barley flour pasting properties, with most of them being unreported in our previous study using a different DH population. The relationships between malt extract and pasting properties were confirmed through QTL mapping. Improved pasting properties can be achieved by pyramiding these QTL using closely linked markers. The fact that different QTL were identified from a different population opens up an opportunity of pyramiding favorite QTL from different varieties (for example Franklin and Naso Nijo) to improve pasting properties.

## Figures and Tables

**Table 1:** Mean and range of pasting properties tested in different environments.

Trait	Environment	TX9425	Naso Nijo	DH	
				Mean ± SD	Range
PV	HZ07	4100	4441	4429 ± 297	3520–5039
	HZ10	4590	4854	4533 ± 233	3793–5139
	YC07	4432	4492	4362 ± 162	3675–4844
	YC10	4158	4347	4241 ± 189	3723–4703
TR	HZ07	2095	2294	2230 ± 183	1551–2579
	HZ10	2150	2268	2107 ± 151	1514–2451.5
	YC07	2064	2164	2032 ± 102	1659–2325
	YC10	1962	2025	1969 ± 126	1548–2270
BD	HZ07	2005	2147	2220 ± 188	1781–2737
	HZ10	2439	2505	2426 ± 170	2079–2924
	YC07	2160	2187	2332 ± 82	2145–2519
	YC10	2195	2322	2271 ± 93	2037–2523
FV	HZ07	3737	4005	3904 ± 269	2895–4444
	HZ10	3718	3811	3627 ± 229	2691–4039
	YC07	4327	4063	4014 ± 158	3517–4535
	YC10	3846	3635	3577 ± 175	2944–4000
SB	HZ07	1657	1659	1677 ± 117	1256–2016
	HZ10	1539	1572	1518 ± 93	1177–1717
	YC07	1946	2216	1986 ± 124	1646–2522
	YC10	1745	1749	1599 ± 101	1341–1927
TTPV	HZ07	6.02	5.98	5.95 ± 0.08	5.70–6.13
	HZ10	5.92	5.92	5.95 ± 0.08	5.73–6.13
	YC07	6.03	6.03	6.05 ± 0.06	5.90–6.20
	YC10	6.03	5.93	5.92 ± 0.09	5.73–6.13
PT	HZ07	79.74	77.22	77.52 ± 1.03	75.40–82.73
	HZ10	77.04	76.42	76.81 ± 0.88	75.00–79.15
	YC07	76.91	75.47	76.37 ± 0.84	68.00–78.70
	YC10	76.83	76.30	76.17 ± 0.66	74.65–77.90

SD: standard deviation; DH: doubled-haploid; PV: peak viscosity; TR: trough; BD: breakdown; FV: final viscosity; SB: setback; TTPV: time to peak viscosity; PT: pasting temperature. HZ: Hangzhou, Zhejiang Province; YC: Yancheng, Jiangsu Province; 07: 2007–2008 growing season; 10: 2010–2011 growing season.

**Table 2:** ANOVA of different pasting properties.

Source of Variation	PV	TR	BD	FV	SB	TTPV	PT
Block	13.89 **	29.56 **	49.28 **	45.65 **	5.49 **	0.67	0.08
Genotype (G)	9.8 **	9.25 **	3.01 **	16.04 **	6.52 **	3.05 **	2.78 **
Location (L)	111.39 **	953.08 **	3.60	3321.46 **	1768.62 **	113.41 **	1.98
Year (Y)	2.25	303.93 **	105.67 **	21.55 **	3296.92 **	224.2 **	362.96 **
Y × L	34.78 **	37.04 **	371.52 **	197.0 **	637.9 **	203.32 **	112.0 **
G × Y	1.15	1.15	1.54 **	1.29 *	0.93	1.59 **	1.34 **
G × L	1.22	1.97 **	2.14 **	3.62 **	2.58 **	1.92 *	1.40 **
G × L × Y	0.96	1.24 *	1.72 **	1.57 **	1.39 **	1.05	1.36 **

* Significant at 5%-level, ** significant at 1%-level. Abbreviations for traits are given in Table 1.

**Table 3:** Quantitative trait loci (QTL) for pasting properties and malt extract (ME) in the DH population of Naso Nijo × TX9425 based on average data from four different environments.

Trait	Linkage Group	QTL Name	Nearest Marker	Position (cM) *	LOD	R^2^ (%)	Additive Effect	Source of Positive Effect	Malt Extract as Covariate	
									LOD	R^2^ (%)
PV	2H	*QPv.NaTx-2H*	7213364S2	43.9	6.39	15.2	69.8	NN	ns	ns
	5H	*QPv.NaTx-5H*	100000433D5	163.9	3.76	8.5	64.5	TX	ns	ns
	1H	*QPv.NaTx-1H*	4170979D1	65.7	3.29	7.4	49.8	TX	ns	ns
TR	2H	*QTr.NaTx-2H*	3267579D2	55.6	8.69	21.3	−59.6	NN	4.36	12.2
	5H	*QTr.NaTx-5H*	100000433D5	163.9	4.51	10.3	50.5	TX	ns	ns
	7H	*QTr.NaTx-7H*	3262448D7	140.4	3.28	7.4	−34.0	TX	ns	ns
BD	2H	*QBd.NaTx-2H*	9772745S2	10.9	6.92	19.5	42.5	NN	ns	ns
FV	2H	*QFv.NaTx-2H*	3267579D2	55.6	7.49	18.4	−85.7	NN	3.98	11.5
	7H	*QFv.NaTx-7H*	3262448D7	140.4	4.12	9.6	−59.9	TX	3.14	8.9
	5H	*QFv.NaTx-5H*	100000433D5	163.9	3.85	8.9	72.4	TX	ns	ns
SB	1H	*QSb.NaTx-1H*	3261249S1	47.5	8.26	19.2	38.2	NN	6.28	17.9
	2H	*QSb.NaTx-2H*	3986974D2	40.8	4.89	10.8	−30.2	NN	3.05	9.1
	7H	*QSb.NaTx-7H*	3262448D7	140.4	3.37	7.3	−23.9	TX	ns	ns
TTPV	2H	*QTtpv.NaTx-2H*	3432484D2	18.9	4.76	11.8	0.017	TX	2.96	8.0
	7H	*QTtpv.NaTx-7H*	3269237D7	140.9	3.75	9.2	0.015	TX	5.11	13.4
PT	2H	*QPt.NaTx-2H*	6429430S2	26.2	8.83	23.2	0.27	TX	5.17	13.8
	1H	*QPt.NaTx-1H*	Bmag0347	74.1	2.92	7.0	−0.15	TX	ns	ns
ME	2H	*QMe.NaTx-2H*	3433862D2	15.4	25.02	53.9	−0.82	NN		

The position is that of the nearest marker; R^2^ means percentage genetic variance explained by the nearest marker; abbreviations for traits are given in Table 1; ns means not significant.

**Table 4:** Correlations between pasting properties and malt extract.

	PV	TR	BD	FV	SB	TTPV	PT	ME
PV	1.00							
TR	0.84 **	1.00						
BD	0.74 **	0.26 **	1.00					
FV	0.78 **	0.93 **	0.22 **	1.00				
SB	0.51 **	0.62 **	0.12	0.86 **	1.00			
TTPV	0.10	0.34 **	−0.25 **	0.34 **	0.27 **	1.00		
PT	−0.40 **	−0.36 **	−0.27 **	−0.43 **	−0.43 **	0.33 **	1.00	
ME	0.42 **	0.21 *	0.50 **	0.13	0.00	−0.31 **	−0.28 **	1.00

* Significant at 5%-level, ** significant at 1%-level. Abbreviations for traits are given in Table 1.

**Table 5:** QTL analysis for malt extract using different pasting properties as covariates.

Trait-Covariate	Chromosome	Nearest Marker	Position (cM)	LOD	R^2^ (%)
ME	2H	3433862D2	15.4	25.02	53.9
ME-PV	2H	3433862D2	15.4	24.8	46.6
ME-TR	2H	3433862D2	15.4	27.0	55.1
ME-BD	2H	3433862D2	15.4	19.0	34.2
ME-FV	2H	3433862D2	15.4	26.1	54.9
ME-SB	2H	3433862D2	15.4	24.7	53.7
ME-TTPV	2H	3433862D2	15.4	21.7	44.9
ME-PT	2H	3433862D2	15.4	25.6	53.2

Abbreviations for traits are given in Table 1.

## Supplementary Materials

The following are available online at http://www.mdpi.com/1422-0067/19/11/3559/s1. Figure S1: QTL for pasting properties QTL (in green) and malt extract (in red). For greater clarity, only selected markers and closest markers to different QTL were shown.
