# Supplementary figures and images for: QTL Mapping Reveals the Relationship between Pasting Properties and Malt Extract in Barley
Source: Int J Mol Sci. 2018 Nov 12;19(11):3559. doi: 10.3390/ijms19113559 (PMC6275068; doi:10.3390/ijms19113559)

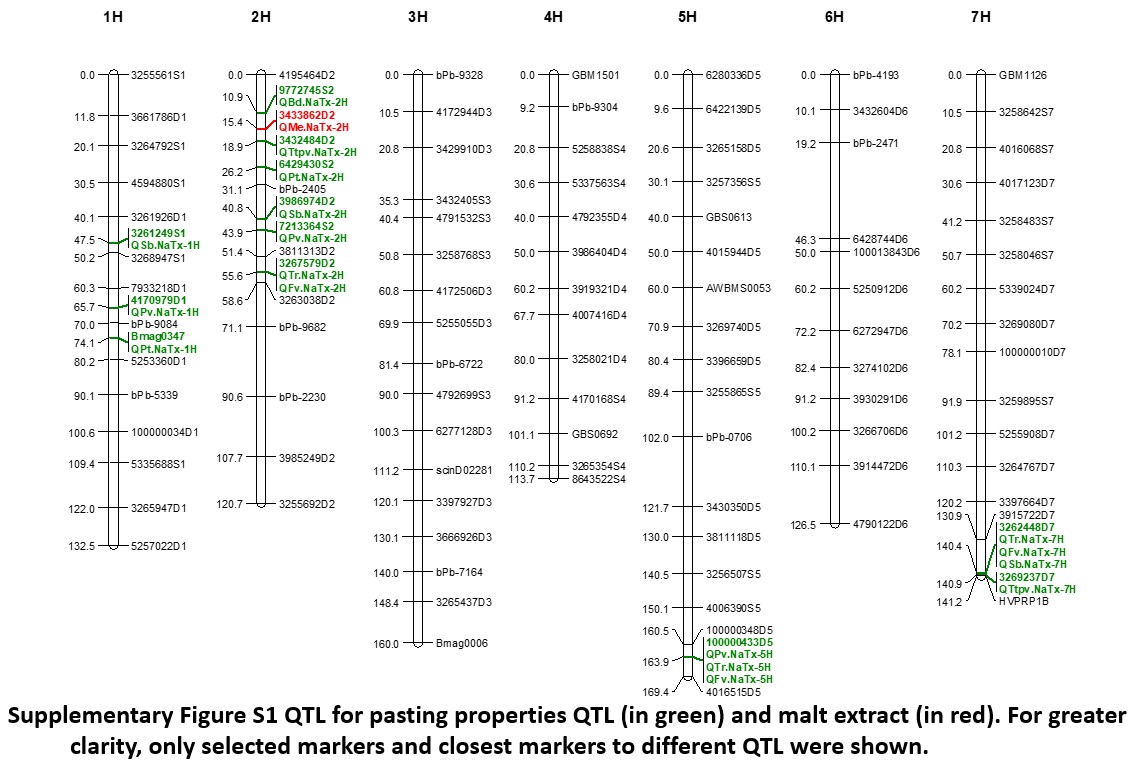

Supplement: Supplementary file 1 [file ijms-19-03559-s001.zip › ijms-362088-SI.jpg]
